# Real-World Long-Term Outcomes of First-Line Pembrolizumab in Advanced PD-L1 ≥ 50% NSCLC: A Systematic Review and Meta-analysis

**DOI:** 10.1245/s10434-026-19138-7

**Published:** 2026-02-05

**Authors:** Guilherme Franceschini Machado, Iago T. C. Grillo, Paula Duarte D’ambrosio, Victoria Trasatti Romao, Lorena Escalante Romero, Tulio Caldonazo, Felipe S. Passos

**Affiliations:** 1 San Paolo Hospital, São Paulo, Brazil; 2 University of Buenos Aires, Buenos Aires, Argentina; 3 Universidade de Sao Paulo, São Paulo, Brazil; 4 Faculdade Anhembi Morumbi, São Paulo, Brazil; 5 GRAACC Hospital, Universidade Federal de São Paulo, São Paulo, Brazil; 6 Department of Cardiothoracic Surgery, Jena University Hospital, Jena, Germany; 7 Department of Cardiothoracic Surgery, Weil Cornell Medicine, New York, NY USA; 8 Materdei Hospital, Salvador, Brazil

**Keywords:** Non-small cell lung cancer, Pembrolizumab, PD-L1 ≥ 50%, Real-world evidence, Overall survival, Progression-free survival, Immune-related adverse events; meta-analysis

## Abstract

**Background:**

Pembrolizumab monotherapy is the standard first-line treatment for advanced non-small-cell lung cancer (NSCLC) with programmed cell death ligand-1 (PD-L1) expression ≥ 50%. However, long-term effectiveness and safety in real-world populations remain underexplored.

**Methods:**

We systematically searched PubMed, Embase, and the Cochrane Library through February 2025 for real-world studies reporting outcomes of first-line pembrolizumab monotherapy in patients with advanced NSCLC and PD-L1 ≥ 50%, excluding those with EGFR/ALK alterations. Primary outcomes were pooled mean overall survival (OS), timepoint-specific OS rates, and progression-free survival. Secondary outcomes included adverse event rates and hazard ratios (HRs) for OS based on age, Eastern Cooperative Oncology Group performance status, PD-L1 intensity, and brain metastases. Kaplan–Meier curves were digitally reconstructed using R.

**Results:**

In total, 12 studies encompassing 17,506 patients were included. The pooled mean OS was 21.0 months (95% confidence interval [CI] 16.9–25.1), and the 60-month OS rate was 29.0%. Mean progression-free survival was 8.7 months (95% CI 6.3–11.0). Any-grade and grade ≥3 adverse events occurred in 52% and 12% of patients, respectively. Age ≥70 years was associated with worse OS (HR 1.26; 95% CI 1.23–1.29). Eastern Cooperative Oncology Group status ≥2 was also linked to poorer outcomes (HR 2.05; 95% CI 1.04–4.05). No significant OS difference was observed for PD-L1 or brain metastases.

**Conclusions:**

Real-world evidence confirms the long-term effectiveness and safety of pembrolizumab monotherapy for advanced NSCLC with PD-L1 ≥50%. Survival outcomes closely mirrored those from previous trials, supporting the generalizability of pembrolizumab’s benefit across routine practice.

**Supplementary Information:**

The online version contains supplementary material available at 10.1245/s10434-026-19138-7.

Lung cancer is considered one of the most commonly diagnosed cancers worldwide^1,2^, comprising 12.4% of all total cases of cancer, and the leading cause of cancer death.^1^ Lung neoplasm is also the leading cause of death in the United States, accounting for 226,650 new cases and 124,730 deaths in 2025. Non-small-cell lung cancer (NSCLC) accounts for approximately 87% of all lung neoplasm cases. ^3^ Over the last few years, advancements in immunohistochemistry studies have enabled the development of targeted therapies for selected subgroups, primarily those without EGFR and ALK mutations. This clinical progress in research has allowed treatment with immunotherapy to become a crucial component of NSCLC treatment. ^4^

The programmed death-ligand 1 (PD-L1), a protein expressed in tumor cells, binds to the programmed cell death 1 (PD-1) receptors on activated T cells, enabling an escape from immune detection. Pembrolizumab is a humanized monoclonal antibody approved by the US Food and Drug Administration that targets PD-1, disrupts the interaction, and reactivates T–cell–mediated cytotoxicity in the treatment of NSCLC.^5,6^ The tumor proportion score (TPS) of PD-L1 expression has emerged as a predictive biomarker, with the ≥ 50% TPS subgroup deriving the most pronounced benefit from pembrolizumab monotherapy.^7^ As a result, longer progression-free survival (PFS) and overall survival (OS) rates were observed when compared with the previously chemotherapy-based treatment.^7,8^

The KEYNOTE-024 central intervention was pembrolizumab as first-line therapy, compared directly with previous standard platinum-based chemotherapy. It improved the median OS of 26.3 months^7^ versus 14.2 months versus chemotherapy alone, along with a superior 5-year OS of 31.9% in patients receiving pembrolizumab as first-line treatment for advanced NSCLC with TPS ≥50% and no EGFR or ALK mutation, which was supported by KEYNOTE-042.^7,9^ This instituted a new setting of therapy without chemotherapy that demonstrated durability and clinical relevance in the previously mentioned population.^8–11^

Although case series support the findings of the former trials,^7,9^ outcome discrepancies between these observational real-world evidence (RWE) studies and the trial raise essential questions about how largely dependent they are on the TPS^10^ and how the strict selection excludes a prevalent population of our clinical practice routine. As a result, the gap between trial efficacy and real-world clinical effectiveness necessitates the synthesis of RWE^12,13^ to assess external validity and therapeutic impact, thereby informing our decision-making in daily practice with real-world data. ^12,14^

We conducted a systematic review and meta-analysis of real-world studies on first-line pembrolizumab monotherapy in advanced NSCLC with PD-L1 ≥50%. Our objectives were to synthesize survival outcomes (including OS rates at various timepoints and mean OS/PFS), explore prognostic factors through subgroup analyses (such as age and PD-L1 levels), characterize adverse events (AEs) (by system and grade), and compare these findings with pivotal clinical trial data, emphasizing the safety gap after longer follow-up comparisons.

## Materials and Methods

### Protocol and Registration

The current systematic review with meta-analysis was registered in the International Prospective Register of Systematic Reviews and Clinical Trials (PROSPERO) under the protocol CRD420251035417. The study was designed in accordance with the Cochrane Handbook for Systematic Reviews of Interventions and the Preferred Reporting Items for Systematic Reviews and Meta-Analyses (PRISMA) 2020 Statement Guidelines^15^ and checklist (Supplemental Figure 1-S3).

**Fig. 1:**
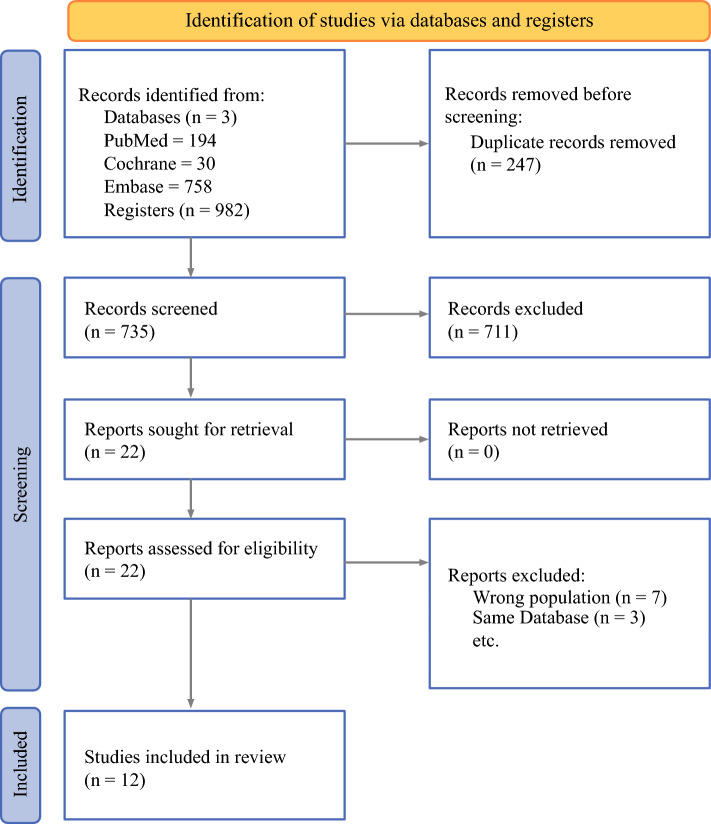
Flow diagram of study selection summarizing study identification, screening, eligibility, and inclusion according to preferred reporting items for systematic reviews and meta-analyses (PRISMA) 2020 guidelines

### Eligibility Criteria

Studies were included if they reported (1) RWE data on (2) adults aged ≥18 years with advanced NSCLC (> 75% with stage III or IV), (3) PD-L1 expression ≥ 50% or unknown due to untested status at treatment initiation, (4) Eastern Cooperative Oncology Group (ECOG) performance status 0–2, or at least 75% of patients with ECOG < 3 or unknown, (5) absence of known EGFR or ALK mutations, and (6) use of first-line pembrolizumab monotherapy.

Patients with PD-L1 expression <50%, ECOG performance status 3–4, known EGFR or ALK mutations, and non-NSCLC histologies were excluded. We excluded studies involving experimental therapies, interventional or randomized designs, protocols, abstracts, editorials, letters to the editor, reviews, systematic reviews, and meta-analyses and non-peer-reviewed sources and non-English data.

### Search Strategy

We systematically searched PubMed, Embase, and the Cochrane Library from inception to February 2025 using the following search terms: “(NSCLC OR “non-small cell lung cancer” OR “lung adenocarcinoma” OR “lung squamous cell carcinoma”) AND (pembrolizumab OR Keytruda OR “anti-PD-1”) AND (monotherapy OR “first-line” OR frontline) AND real”) Supplemental Figure 4.

The references of all included studies were also manually searched to identify additional eligible studies. Two authors (G.F.M. and V.T.R.) independently performed the screening process using Rayyan software 1.6 (Cambridge, Massachusetts, USA). Baseline characteristics and outcome data were extracted independently by two authors (L.E.R. and G.F.M.). Any discrepancies were resolved through consensus.

### Primary and Secondary Outcomes

This review and meta-analysis assessed primary outcomes of OS, reported as pooled mean OS and rates at 12, 24, 36, 48, and 60 months, and PFS, as pooled mean PFS. Secondary outcomes included pooled rates of AEs and grade ≥ 3 AEs by system (e.g., skin, endocrinological) and hazard ratios (HRs) for OS based on factors such as age, ECOG, PD-L1 levels, and brain metastasis.

All included studies defined the index exposure as first-line pembrolizumab, administered as monotherapy. OS was consistently defined as the time from initiation of first-line pembrolizumab to death from any cause, without censoring at the start of subsequent therapies. PFS, when reported, was defined uniformly across studies as the time from pembrolizumab initiation to disease progression or death, with censoring at the introduction of a new systemic therapy or at the last available follow-up. Multiple cohorts documented post-progression treatment patterns, with 21–37% of patients receiving second-line systemic therapy, most commonly platinum-based chemotherapy, non-platinum cytotoxic agents, or less frequently, immune checkpoint inhibitors.

### Subgroup Analysis

For survival outcomes, we performed meta-analyses to estimate pooled OS rates at 12, 24, 36, 48, and 60 months. Studies reporting the survival probability or data for each time point were included in separate meta-analyses. For prognostic factors, we meta-analyzed HRs and 95% confidence intervals (CIs) for OS comparing subgroups based on age and PD-L1 levels (e.g., very high vs. high, PD-L1 ≥ 50%). Meta-analyses of AE rates were done for overall AEs, grade ≥ 3 AEs, and common AEs by system (e.g., skin, endocrinological).

When outcomes were presented as medians or ranges, means and standard deviations were estimated using validated statistical methods.^17,18^

### Sensitivity Analyses

A leave-one-out (LOO) sensitivity analysis was conducted for meta-analyses to assess the influence of individual studies on the pooled effect estimates and heterogeneity. This involved iteratively removing one study at a time and recalculating the pooled HR using R.^16^

### Quality Assessment

The risk of bias was evaluated using the Risk of Bias in Non-randomized Studies of Interventions (ROBINS-I) tool, which is specifically designed for observational studies. Two independent reviewers (G.F.M. and V.T.R.) performed the assessments, and discrepancies were resolved through a consensus process. To evaluate the potential for bias attributable to missing results (publication bias), funnel plots were generated for the primary outcomes and subsequently scrutinized visually for asymmetry. Furthermore, Egger’s regression test was employed to assess small-study effects quantitatively when 10 or more studies were available in a synthesis.

### Statistical Analysis

Single-arm and comparative meta-analyses were conducted using R version 4.4.1. Random-effects models (DerSimonian–Laird method, with Hartung–Knapp adjustment for HR analyses when appropriate) were used for all syntheses to account for anticipated between-study heterogeneity.

Published Kaplan–Meier curves for OS were digitized using WebPlotDigitizer (v4.6) to extract time–survival coordinate pairs when point estimates were not available or to supplement reported data. Corresponding numbers at risk at specified time intervals were also collected. Survival values were ensured to be monotonically non-increasing. This digitized data, along with numbers at risk, was used to reconstruct a pooled real-world OS curve for illustrative comparison with benchmark trial data, using the methods described elsewhere by Guyot et al.^19^ for deriving pseudo-individual patient data, also in R.

For proportions (OS rates at specific time points, AE rates), a generalized linear mixed model using a logit transformation was employed, with between-study variance (*τ*^2^) estimated using the restricted maximum likelihood method. Pooled proportions with 95% CIs were calculated. For HRs (OS by age, OS by PD-L1), log(HR) values and their standard errors were pooled, and the results were back-transformed to the HR scale.

Heterogeneity was assessed using Cochran's Q test (p-value), the I^2^ statistic to quantify the proportion of total variation due to between-study differences, and the τ^2^ statistic for absolute between-study variance. Forest plots were generated for visualization, typically using the RevMan5 layout.

## Results

### Study Selection and Characteristics

The initial search yielded 982 results, as described in Fig. 1. After removing 247 duplicate records and applying the inclusion and exclusion criteria, 22 studies were thoroughly reviewed. A total of 12 real-world studies met our inclusion criteria, encompassing a total of 17,506 patients. Seven studies were excluded because of incorrect population inclusion, and three studies were removed because of overlapping populations. Table 1 summarizes the baseline characteristics of the included populations.

**Table 1:** Baseline characteristics of included patients

Author, year	Location, recruitment period	Sample (n)	Male (%)	Age (median)	Positive smoking history (%)	NSCLC histology type (%)	Metastases: site (%)	ECOG: class (%)
Velcheti et al.^28^ 2024	USA, 2016–2020	804	49.8	72	93	NSq: 69 Sq: 26 NOS: 5	CNS (11.4)	0–1 (100)
Tambo et al.^27^ 2025	Japan, 2015–2018	95	74.7	72	81.1	Ad 62.1 AdSq: 1 Sq: 32.6 Other: 4.3	< 3 sites (80) ≥ 3 sites (20)	0–1 (77.9) 2 (11.6)
Cvijic,^20^ 2023	Croatia, 2018–2021	78	58	63	47	Ad: 71 AdSq: 1 Sq: 13 NOS: 10 PD: 5	CNS (23)	0–1 (100)
Cortellini et al.^24^ 2025	14 countries (Europe, USA, and Brazil), 2015–2018	1050	59.8	69	100	Sq: 22.8 Ad: 73.5 NOS: 3.7	CNS (19.9) ≤ 3 sites (90.1) > 3 sites (9.9)	0–1 (81.8) 2-4 (15.3) Unknown (2.9)
Cafaro et al.^23^ 2024	Italy, 2017–2022	880	70.2	69.9	80.6	Ad: 76.5 NOS: 4.2 LC: 0.5 AdSq: 0.8 Sq: 18 NOS: <0.01	CNS (15.7)	0 (42) 1 (49.9) 2 (8.1)
Decroisette et al.^29^ 2024	France, 2017–2019	845	67.8	65	90.4	NSq: 73.2	CNS (20.8)	0–1 (78.1) 2–4 (21.9)
Faoro et al.^21^ 2023	Italy, 2020	98	64.3	73	NR	NR	CNS (10.2)	0 (25.5) 1 (65.3) 2 (9.2)
Gadgeel et al.^25^ 2024	USA, 2016–2018	505	48.9	71.2	84.6	Sq: 18.6 Nsq: 65.1 Other: 1.6	CNS (12.7)	0–1 (61.2) 2 (22.6)
Goto et al.^22^ 2022	Japan, 2017–2018	441	78.5	70	90	Nsq (63.6) Sq (29.3) Other (7.1)	CNS (20.9)	0 (19.3) 1 (35.1) 2 (8.2)
Jimenez Galan,^31^ 2023	Spain, 2017–2020	101	74.3	67	92.1	AdSq (68.3) Sq (18.8) PD (8.9) Others (4.0)	CNS (15.8)	0 (19.8) 1 (42.6) 2 (29.7) 3 (7.9)
Rousseau et al.^30^ 2024	France, 2015–2022	44539	67	65	NR	NR	NR	NR
Shah et al.^26^ 2022	USA, 2016–2021	196	46	68	94	Nsq (70)	NR	NR
KEYNOTE-024^7^		154	60	65	96.8	Sq (19) NSq (81)	CNS (11.7)	0–1 (99) 2–4 (1)

Ad, adenocarcinoma; AdSq, adenosquamous; CNS, central nervous system; LC, large-cell; NOS, not otherwise specified; NSCLC, non-small-cell lung cancer; NSq, non-squamous; PD, poorly differentiated; Sq, squamous

During data synthesis for specific outcomes, some studies or data points were excluded if they did not meet the inclusion criteria for pooling. For example, one study^20^ reported some events exceeding the number of patients at risk in at least one follow-up time point for OS rates, and these specific data points were excluded from the respective time-point analyses. Additionally, for pooled median OS, one study^21^ was excluded from this analysis because data were insufficient to estimate the standard deviation, and one study^22^ did not report median OS in a usable format for this specific synthesis. Data extraction table, coding, and material are available upon request.

### OS (Mean and Timepoint Rates)

**Fig. 2:**
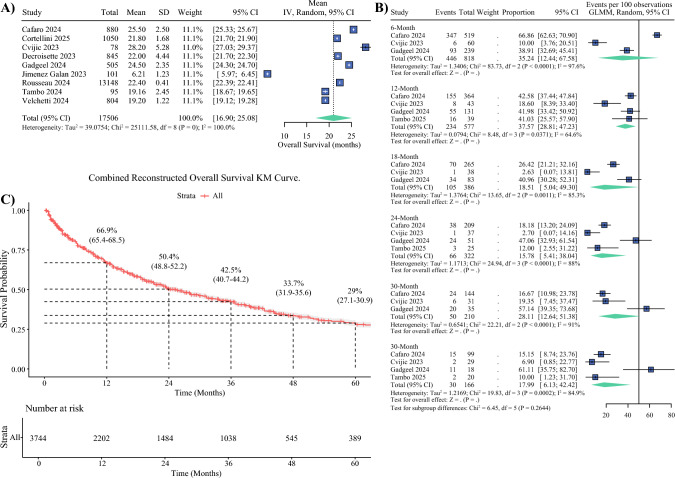
Overall survival outcomes in real-world cohorts. **A** Forest plot of pooled mean overall survival (OS) across nine cohorts, with study-level estimates ranging from 6.21 to 28.20 months. The pooled mean OS was 21.0 months (95% confidence interval [CI] 16.9–25.1). **B** Forest plot of pooled OS rates at fixed time points (12, 24, 36, and 48 months), showing survival probabilities declining over time. **C** Reconstructed pooled Kaplan–Meier (KM) curve for OS derived from digitized data (n = 3744 patients), benchmarked against clinical trial outcomes, with estimated 12-, 24-, 36-, 48-, and 60-month survival probabilities. GLMM, generalized linear mixed model; IV, inverse variance; SD, standard deviation

The pooled mean OS across nine real-world cohorts was 21.00 months (95% CI 16.90–25.08; Fig. 2A). Study-level means demonstrated substantial variability, ranging from 6.21 months in the study by Jimenez Galan (2023) to 28.20 months in the cohort reported by Cvijic (2023).

When evaluating OS rates at fixed time points, the pooled 12-month OS rate was estimated at 24.74% (95% CI 12.79–42.09). This proportion increased at 24 months to 35.38% (95% CI 23.51–49.38), remained relatively stable at 36 months at 33.22% (95% CI 19.12–51.22), and declined to 18.48% (95% CI 2.57–68.18) by the 48-month mark. Forest plots corresponding to these time-specific estimates are presented in Fig. 2B.

We reconstructed a pooled Kaplan–Meier survival curve from individual patient-level estimates derived from the published datasets^20,23–28^ depicted in Fig. 2C, which represents OS in real-world settings. At baseline, the pooled population included 3744 patients at risk. The estimated OS probability at 12 months was 66.9% (95% CI 65.4–68.5), with 2202 patients still under observation. At 24 months, the survival rate declined to 50.4% (95% CI 48.8–52.2), with 1484 patients remaining at risk. This trend continued at 36 months, where survival was estimated at 42.5% (95% CI 40.7–44.2), and 1038 patients remained under follow-up. At 48 months, survival probability was 33.7% (95% CI 31.9–35.6) with 545 individuals at risk, and by 60 months, the 5-year OS rate was 29.0% (95% CI 27.1–30.9), with 389 patients remaining at risk.

### PFS

**Fig. 3:**
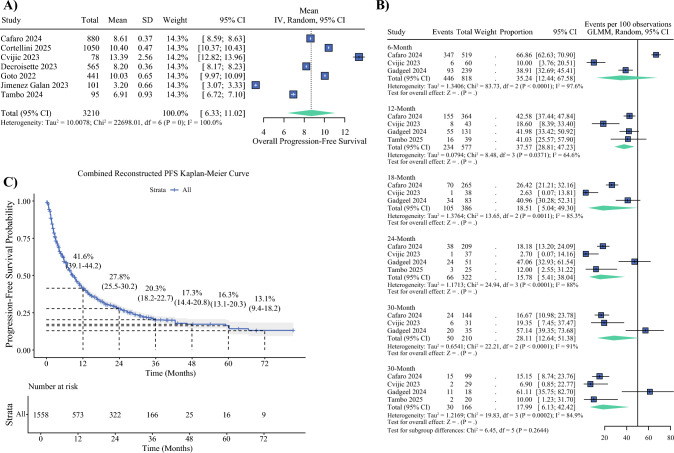
Progression-free survival (PFS) outcomes in real-world cohorts. **A** Forest plot of pooled mean PFS across seven cohorts, with study-level estimates ranging from 3.20 to 13.39 months. The pooled mean PFS was 8.67 months (95% confidence interval [CI] 6.33–11.02). **B** Forest plot of pooled PFS rates at fixed time points (6, 12, 18, 24, and 36 months), showing a consistent decline in progression-free probability over time. **C** Reconstructed pooled Kaplan–Meier curve for PFS derived from digitized data (*n* = 1558 patients), with estimated survival probabilities at 12, 24, 36, 48, 60, and 72 months. GLMM, generalized linear mixed model; IV, inverse variance; SD, standard deviation

The pooled mean PFS from real-world cohorts encompassing 3210 patients was 8.67 months (95% CI 6.33–11.02; Fig. 3A). Study-level means ranged from 3.20 to 13.39 months.

PFS rates at clinically relevant time points were also pooled (Fig. 3B). At 6 months, the pooled PFS rate was 35.24% (95% CI 12.44–67.58). At 12 months, it was 37.57% (95% CI 28.81–47.23), declining to 15.78% (95% CI 5.04–49.30) at 18 months and 15.78% (95% CI 7.41–38.04) at 24 months. By 36 months, the PFS rate was 17.99% (95% CI 6.13–42.42).

Figure 3C presents the reconstructed Kaplan–Meier curve for PFS, derived from pooled real-world data across seven studies.^20,23,25,27^ The analysis began with 1558 patients at risk at time zero. At 12 months, the estimated PFS probability was 41.6% (95% CI 39.1–44.2), with 573 patients still progression free. This rate declined to 27.8% (95% CI 25.5–30.2) at 24 months and 20.3% (95% CI 18.2–22.7) at 36 months. By 48 months, the PFS probability was 17.3% (95% CI 14.4–20.8), followed by 16.3% (95% CI 13.1–20.3) at 60 months and 13.1% (95% CI 9.4–18.2) at 72 months.

The number of patients at risk at each timepoint interval corresponded to 322 at 24 months, 166 at 36 months, 25 at 48 months, 16 at 60 months, and nine at 72 months.

### AEs

**Fig. 4:**
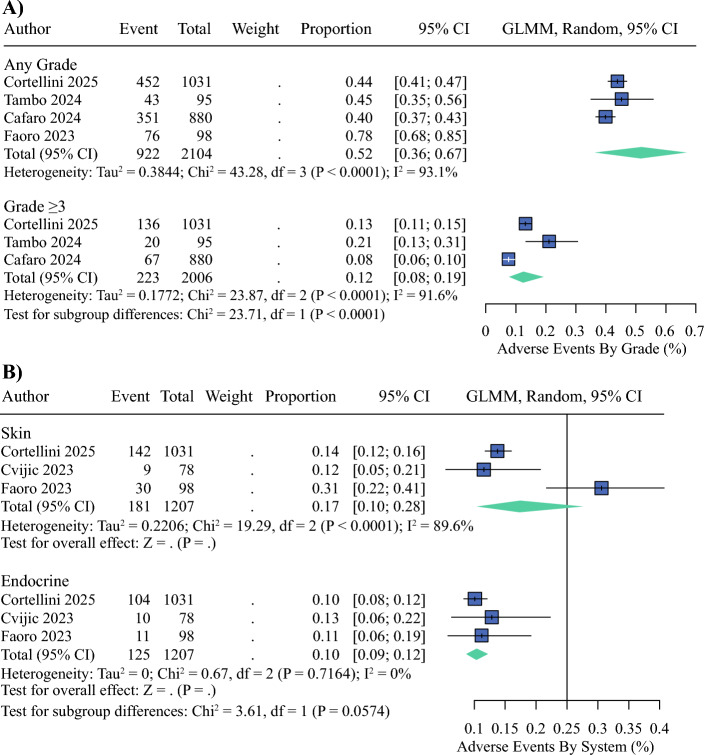
Adverse events in real-world cohorts. **A** Forest plot of pooled incidence of any-grade adverse events (AEs) and grade ≥ 3 AEs across three studies. The pooled rates were 52% (95% confidence interval [CI] 36–67) for any-grade AEs and 12% (95% CI 8–19) for grade ≥ 3 AEs. **B** Forest plot of system-specific AEs, showing the most frequent toxicities: skin-related events (17%; 95% CI 10–28) and endocrinological events (10%; 95% CI 9–12). GLMM, generalized linear mixed model

Three real-world studies^20,21,24^ were pooled, and the incidence of any-grade AE across these cohorts was 52% (95% CI 36–67; Fig. 4A). In contrast, the rate of grade ≥3 AEs was lower, at 12% (95% CI 8–19; Fig. 4A).

The most frequently reported toxicities were skin-related events, with a pooled incidence of 17% (95% CI 10–28).^20,21,24^ Endocrinological toxicities were the next most common, with a pooled rate of 10% (95% CI 9–12) reported consistently across the same studies (Fig. 4B). Notably, skin-related events demonstrated high inter-study heterogeneity (I^2^ = 89.6%), whereas endocrine AEs were consistent across cohorts (I^2^ = 0%).

When stratified by severity, grade ≥3 AEs included serious immune-related events, particularly endocrinopathies and pulmonary complications, although not uniformly specified by system in all datasets.

### OS Subgroup Analyses (Hazard Ratios)

#### Age

**Fig. 5:**
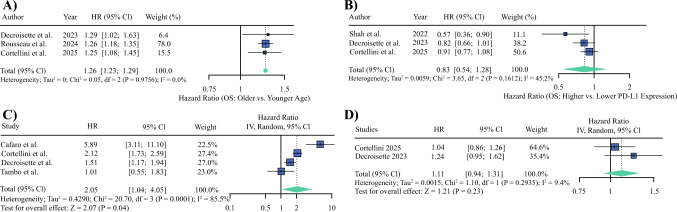
Subgroup analyses of overall survival (OS) hazard ratios (HRs). **A** Age: forest plot of OS comparing patients aged ≥70 years versus younger patients across three studies. The pooled HR was 1.26 (95% confidence interval [CI] 1.23–1.29; *p* < 0.001), indicating significantly poorer outcomes with older age. **B** Programmed cell death ligand-1 (PD-L1) expression intensity: forest plot of OS comparing relatively higher versus lower PD-L1 expression within the ≥50% subgroup. The pooled HR was 0.83 (95% CI 0.54–1.28; *p* = 0.204), with moderate heterogeneity. **C** Eastern Cooperative Oncology Group (ECOG) performance status: forest plot of OS comparing patients with ECOG 0–1 versus ≥2. The pooled HR was 2.05 (95% CI 1.04–4.05; *p* = 0.04), indicating significantly worse survival in patients with reduced performance status. **D** Brain metastases: forest plot of OS comparing patients with versus without brain metastases. The pooled HR was 1.11 (95% CI 0.94–1.31; *p* = 0.230), showing no significant difference. IV, inverse variance

Three studies^24,29,30^ reported HRs comparing patients aged ≥ 70 years against younger patients. The pooled HR for OS was 1.26 (95% CI 1.23–1.29; *p* < 0.001), indicating a statistically significant association between older age and poorer survival outcomes (Fig. 5A). No heterogeneity was detected (I^2^ = 0%, τ^2^ = 0).

#### PD-L1

Three studies^24,26,29^ compared OS among patients with relatively higher versus lower PD-L1 expression within the ≥50% cohort. The pooled HR was 0.83 (95% CI 0.54–1.28; p = 0.204; Fig. 5B). Moderate heterogeneity was observed (I^2^ = 45.2%, τ^2^ = 0.0059). Four studies^23,24,27,29^ compared OS in patients with ECOG 0–1 versus ≥2. The pooled HR was 2.05 (95% CI 1.04–4.05; p = 0.04; Fig. 5C), indicating significantly poorer survival among patients with reduced performance status. Heterogeneity was high (I^2^ = 85.5%, τ^2^ = 0.4290). Two studies^24,29^ reported HRs comparing patients with versus without brain metastases. The pooled HR was 1.11 (95% CI 0.94–1.31; p = 0.230; Fig. 5D). Heterogeneity was low (I^2^ = 9.4%, τ^2^ = 0.0015).

#### Sensitivity Analyses

##### Mean OS

The overall pooled mean OS was 20.99 months (95% CI 16.90–25.08). When omitting one study at a time, estimates ranged narrowly from 20.10 months (omitting Cvijic et al., 2023) to 22.82 months (omitting Jimenez Galan et al. 2023), with overlapping CIs in all cases. This stability indicates that no individual study disproportionately influenced the overall mean survival and that the estimate is resilient to dataset-specific variability (Supplementary Figure 5).

##### Timepoint-Specific OS Rates (6–48 Months)

LOO sensitivity analyses for each key survival milestone further reinforced the robustness of our findings. The 6-month OS pooled rate remained at 33% (95% CI 18–51), with individual estimates ranging from 27% to 42% across iterations in Supplemental Figure 6. At 12 months, the pooled rate was 25% (95% CI 12–45), as shown in Supplemental Figure 7, and the LOO estimates remained within a 0.18–0.31 range. Similar stability was seen at 18 months (22%; 95% CI 14–34), Supplemental Figure 8, and 24 months (20%; 95% CI 11–32), Supplemental Figure 9, showing minimal sensitivity to individual studies such as Cortellini et al.^24^ or Shah et al.^26^

**Fig. 6:**
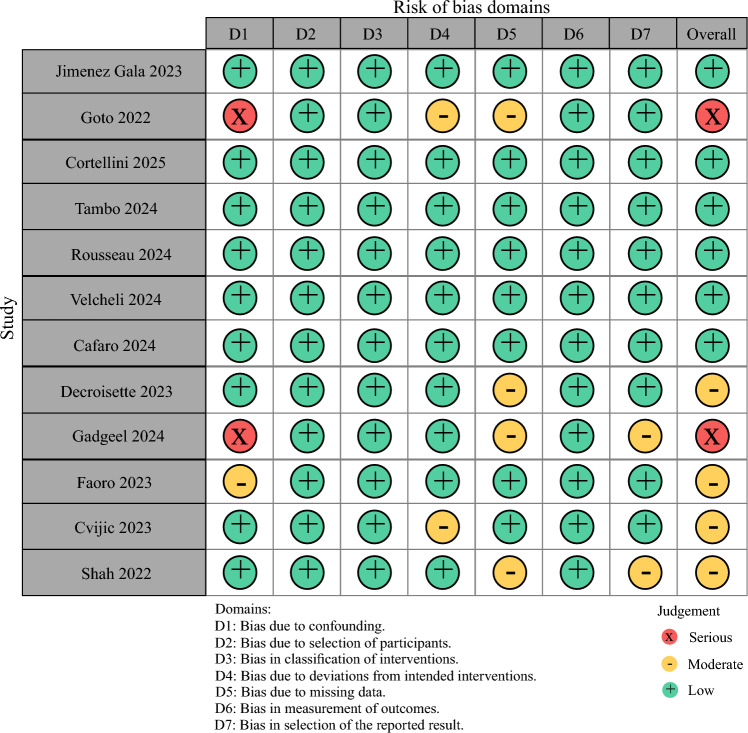
Critical appraisal of studies according to the Risk Of Bias In Non-randomized Studies of Interventions (ROBINS-I) tool

Longer-term timepoints also demonstrated consistent survival probabilities. The 30-month OS remained centered at 18% (95% CI 8–35) in Supplemental Figure 10, whereas the 36-month rate was 23% (95% CI 12–40) across all iterations in Supplemental Figure 11. The pooled estimate was 35% (95% CI 5–84) at 42 months (Supplemental Figure 12) and 29% (95% CI 6–72) at 48 months (Supplemental Figure 13), with slightly wider intervals due to reduced sample size and longer follow-up heterogeneity.

##### PFS

The LOO analysis was 8.67 months (95% CI 6.33–11.02), yielding mean PFS values ranging from 7.89 months (omitting Cvijic et al. 2023) to 9.58 months (omitting Jimenez Galan et al. 2023) (Supplemental Figure 14).

##### OS by PD-L1 Expression (≥50%)

The LOO sensitivity showed modest heterogeneity in the overall model (HR 0.83; 95% CI 0.54–1.28; I^2^ = 45.2%). When excluding individual studies, the pooled HRs remained stable, ranging from 0.73 to 0.87, whereas the I^2^ statistic varied between 0% and 71.6% (Supplemental Figure 15).

##### OS by ECOG

LOO analysis showed a pooled HR of 2.38 (95% CI 1.41–4.01; I^2^ = 81.4%). Exclusion of individual studies resulted in pooled HRs ranging from 1.86 (omitting Cafaro et al.^23^) to 2.84 (omitting Decroisette et al.^29^), with heterogeneity fluctuating between 54.7% and 87.6% (Supplemental Figure 16).

##### Publication Bias

Formal assessment of publication bias was not conducted, in line with Cochrane Handbook recommendations, as fewer than 10 studies were available.

##### Quality Assessment

The results of the risk assessment are summarized in Fig. 6. Nine of the 12 included studies were judged to be at low overall risk of bias, with consistent low-risk ratings across most domains. This reflects the relatively robust methodological design and outcome ascertainment typical of contemporary RWE.

However, two studies (Goto et al.^22^ and Gadgeel et al.^25^) demonstrated a serious risk of bias, primarily due to confounding (D1) and participant selection (D2).

Moderate risk was noted sporadically in domains related to missing data and protocol deviations (e.g., non-standardized treatment intervals and loss to follow-up), particularly in registry-based studies. These limitations are expected in RWE and were addressed, when possible, by extracting survival endpoints from validated electronic health records or published Kaplan–Meier data.

Importantly, no study was deemed to have a critical risk of bias that warranted exclusion.

## Discussion

Our systematic review and meta-analysis of 12 real-world studies support the use of first-line pembrolizumab monotherapy as offering substantial and durable benefits for patients with advanced NSCLC with PD-L1 expression levels of ≥50%. In this pooled cohort, mean OS was 21 months, with a 60-month OS of 29.0%, and only 12% experienced grade ≥3 AEs, reinforcing the external validity of trial data and demonstrating the effectiveness of pembrolizumab in routine practice.

A key finding is the alignment of long-term outcomes with the KEYNOTE-024 trial. We reconstructed Kaplan–Meier curves from real-world data and patient-at-risk tables. The 12-month OS rate was slightly lower (66.9% vs. 73.2%), probably because we included patients with ECOG ≥ 2, who were older, or who had comorbidities that were excluded from trials. Survival rates converged at later timepoints, and OS at 36 months (42.5% vs. 43.7%) and 60 months (29.0% vs. 31.9%) was nearly identical. The 60-month PFS was also similar (16.3% vs. 12.8%), indicating a durable benefit.

Age ≥ 70 years was linked to worse OS (HR 1.26; 95% CI 1.23–1.29), with consistency across studies. ECOG ≥2 also worsened OS (HR 2.05) but with high heterogeneity (I^2^=85.5%), suggesting variability in assessments or practices. PD-L1 levels and brain metastases showed no significant effect, possibly because of threshold effects or limited data. Sensitivity analyses confirmed these results.

Safety results differed from those in KEYNOTE-024: 31.2% had grade ≥ 3 AEs in trials, versus 12% in real-world data. Underreporting or treatment individualization may explain this. Clinicians might avoid immunotherapy in frail patients or manage side effects more conservatively. Overall, pembrolizumab appears well-tolerated in practice, with fewer severe toxicities than expected. We employed comprehensive LOO sensitivity analyses to evaluate the stability of our pooled estimates. Across all survival metrics, including mean OS, mean PFS, and subgroup HRs, LOO iterations yielded consistent results with overlapping CIs. This methodological step confirmed that no single study unduly influenced our conclusions, reinforcing the internal validity and resilience of our findings against the inherent variability in RWE.

This study represents the largest meta-analysis to date evaluating real-world outcomes of first-line pembrolizumab in advanced NSCLC with PD-L1 ≥ 50%, incorporating up to 5 years of follow-up across multiple clinically relevant subgroups. Through advanced analytic approaches, including reconstructed Kaplan–Meier curves and subgroup-specific modeling, we provide granular survival estimates that clinicians can use to benchmark expected patient trajectories in routine practice. The review followed fully transparent RWE and PRISMA-compliant methods, enhancing reproducibility and interpretability. Importantly, because all included cohorts evaluated pembrolizumab exclusively as first-line therapy, PFS primarily reflects the direct therapeutic effect of pembrolizumab, whereas OS captures the broader real-world clinical course, including the influence of subsequent systemic therapies. Across studies, the most prevalent second-line regimen was platinum-based doublet chemotherapy, typically carboplatin plus pemetrexed or carboplatin plus paclitaxel.

Limitations include the observational design, non-standardized PD-L1 definitions (70–90%), inconsistent AE reporting, and small subgroup analyses, which limit power and applicability. Findings show the survival benefit with pembrolizumab persists broadly, and the low severe AE rates indicate tolerability. Future research should focus on standardized real-world data, underrepresented groups, frailty, and quality-of-life outcomes-based decision-making.

Future work should gather harmonized, prospective real-world data, particularly for underrepresented groups, such as those with ECOG scores of ≥ 2 and comorbidities, to enhance immunotherapy strategies and equity in lung cancer care.

## Conclusion

Our meta-analysis shows that the long-term effectiveness, durability, and safety seen in controlled trials with pembrolizumab are also evident in routine practice. It confirms pembrolizumab as a highly effective and tolerable standard for advanced NSCLC with high PD-L1, regardless of comorbidities or performance status. The findings highlight the importance of RWE in guiding cancer care.

## Supplementary Information

Below is the link to the electronic supplementary material.

Supplementary file1 (JPG 1002 KB)

Supplementary file2 (JPG 881 KB)

Supplementary file3 (JPG 481 KB)

Supplementary file4 (PNG 68 KB)

Supplementary file5 (PNG 179 KB)

Supplementary file6 (PNG 53 KB)

Supplementary file7 (PNG 61 KB)

Supplementary file8 (PNG 52 KB)

Supplementary file9 (PNG 65 KB)

Supplementary file10 (PNG 52 KB)

Supplementary file11 (PNG 62 KB)

Supplementary file12 (PNG 53 KB)

Supplementary file13 (PNG 58 KB)

Supplementary file14 (PNG 152 KB)

Supplementary file15 (PNG 110 KB)

Supplementary file16 (PNG 127 KB)
