# Supplementary figures and images for: Real-World Long-Term Outcomes of First-Line Pembrolizumab in Advanced PD-L1 ≥ 50% NSCLC: A Systematic Review and Meta-analysis
Source: Ann Surg Oncol. 2026 Feb 5;33(5):4324–35. doi: 10.1245/s10434-026-19138-7 (PMC13083314; doi:10.1245/s10434-026-19138-7)

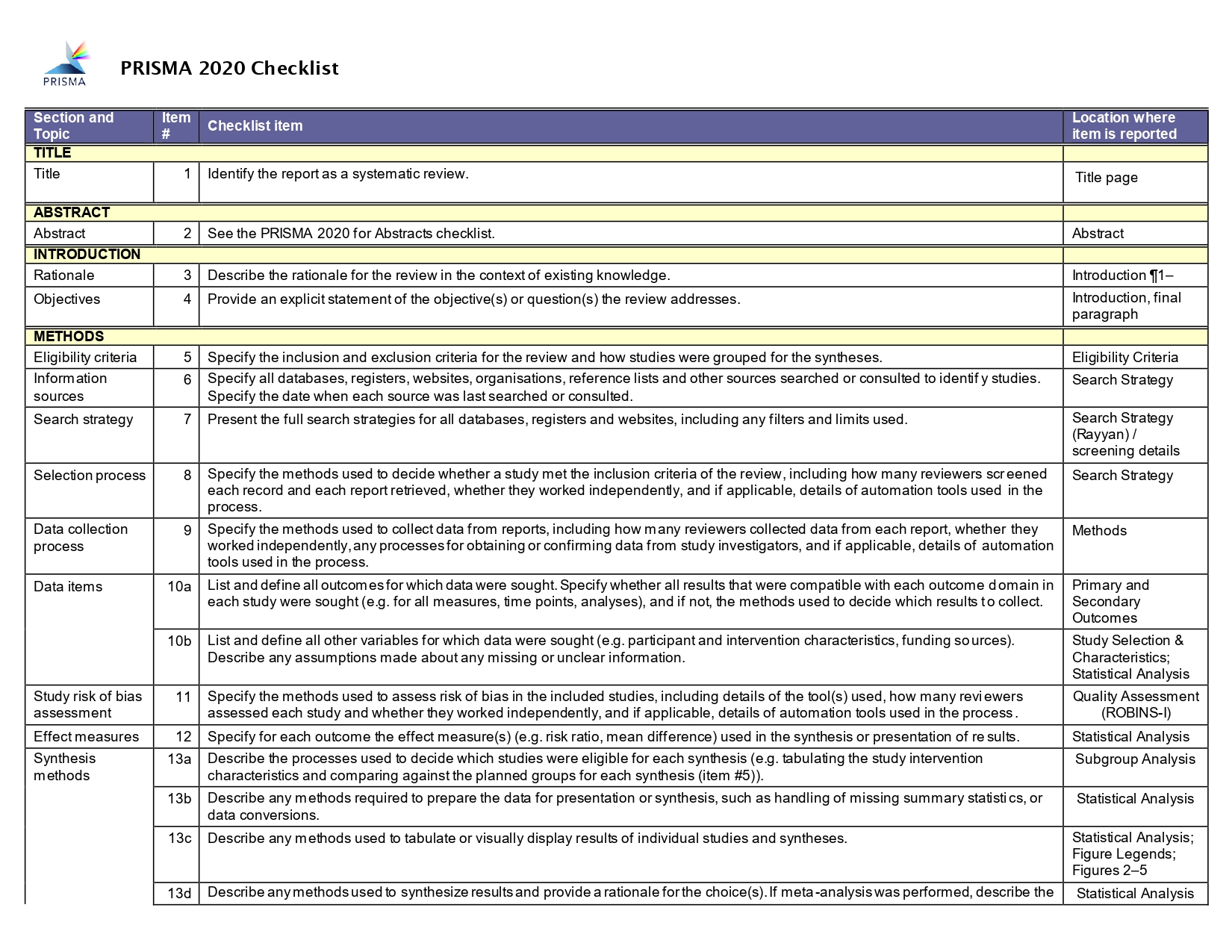

Supplement: Supplementary file 1 — Supplementary file1 (JPG 1002 KB) [file 10434_2026_19138_MOESM1_ESM.jpg]

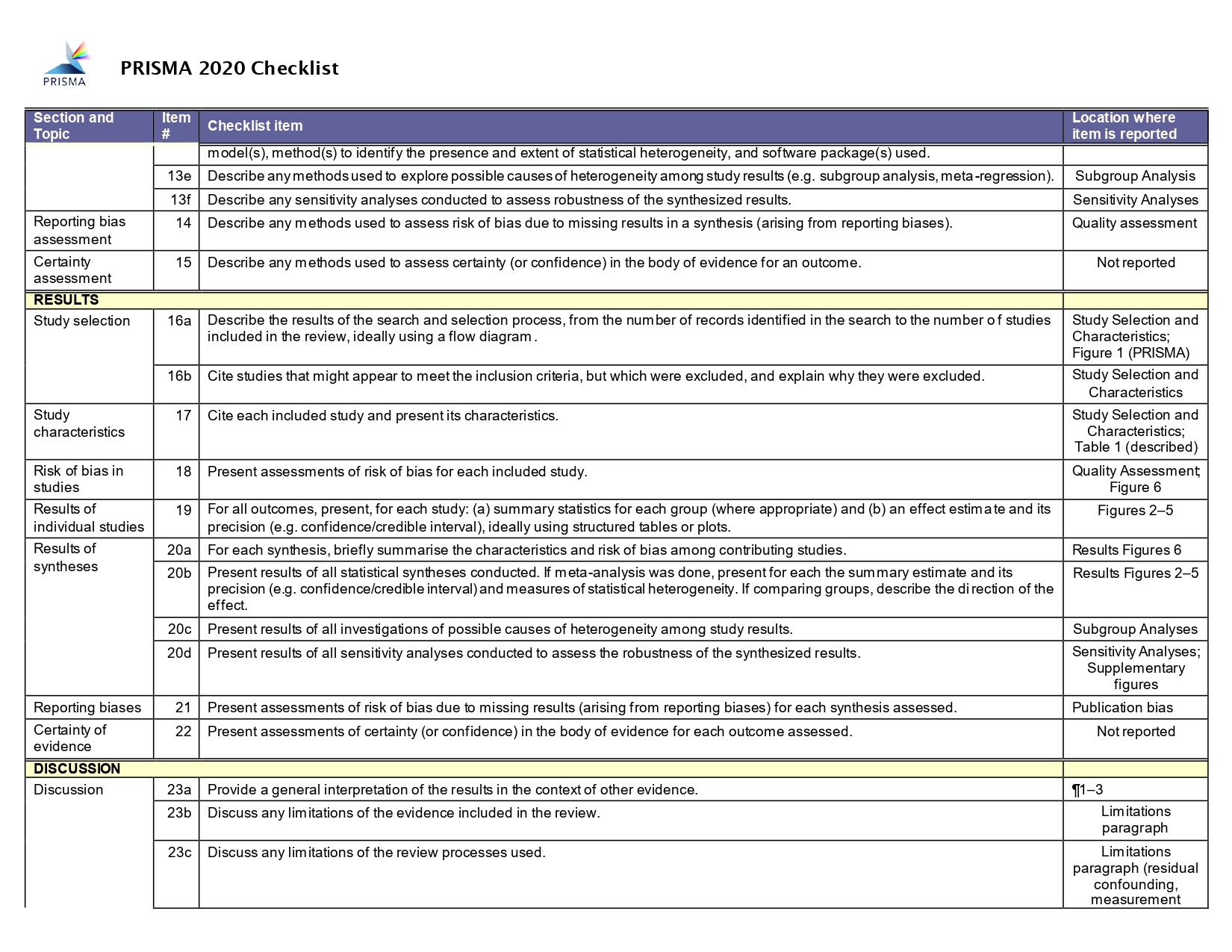

Supplement: Supplementary file 2 — Supplementary file2 (JPG 881 KB) [file 10434_2026_19138_MOESM2_ESM.jpg]

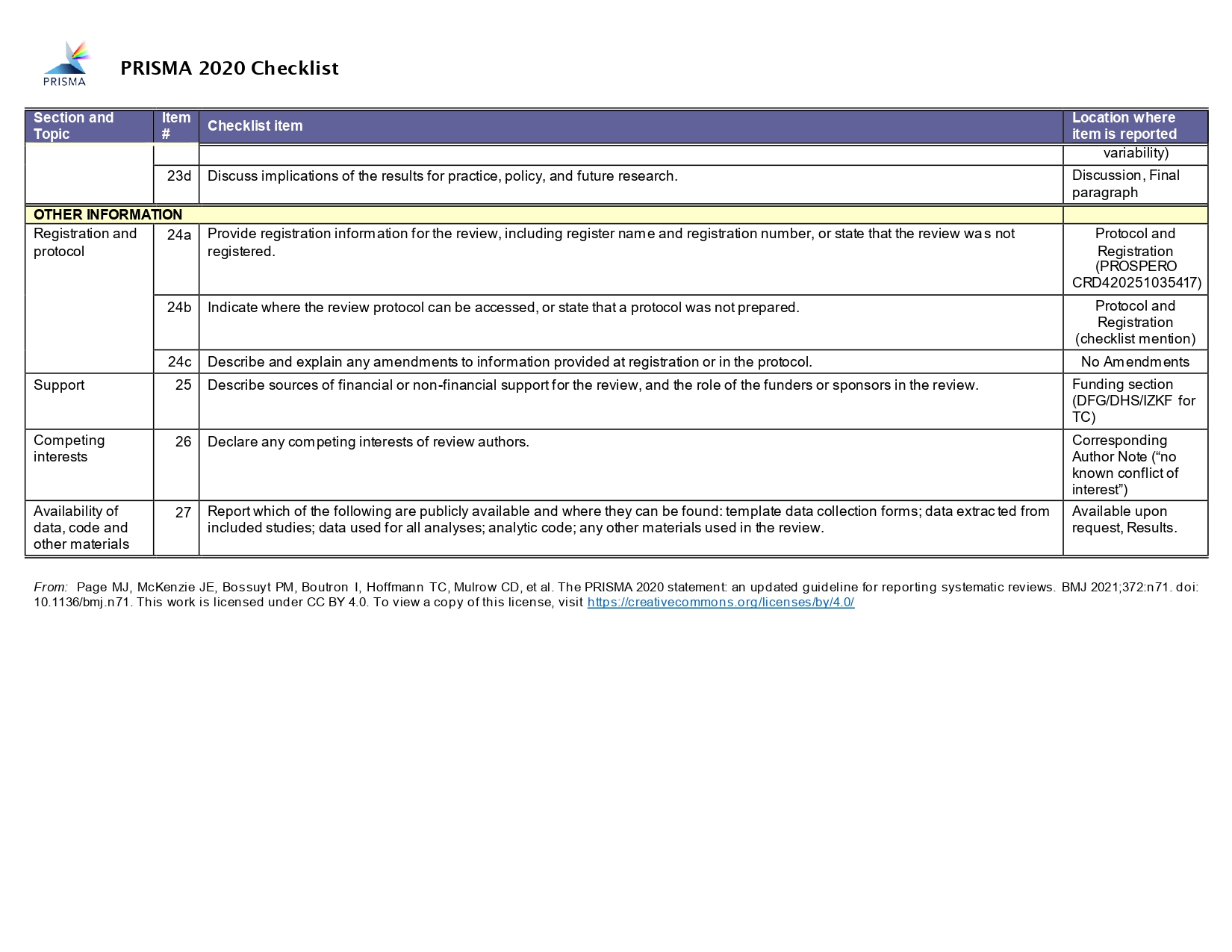

Supplement: Supplementary file 3 — Supplementary file3 (JPG 481 KB) [file 10434_2026_19138_MOESM3_ESM.jpg]

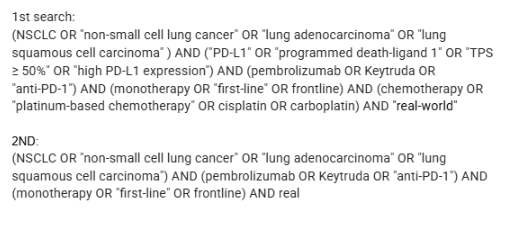

Supplement: Supplementary file 4 — Supplementary file4 (PNG 68 KB) [file 10434_2026_19138_MOESM4_ESM.png]

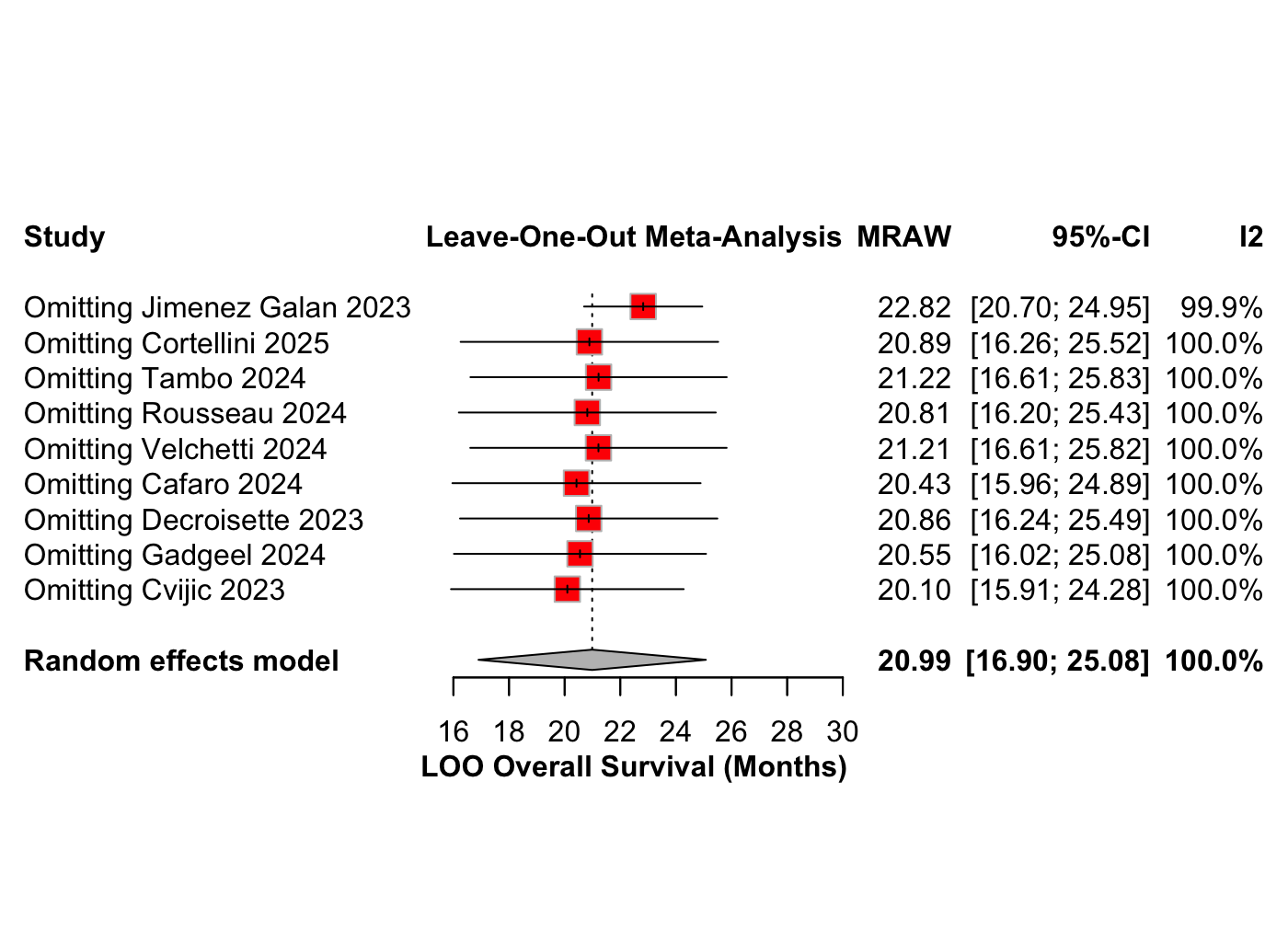

Supplement: Supplementary file 5 — Supplementary file5 (PNG 179 KB) [file 10434_2026_19138_MOESM5_ESM.png]

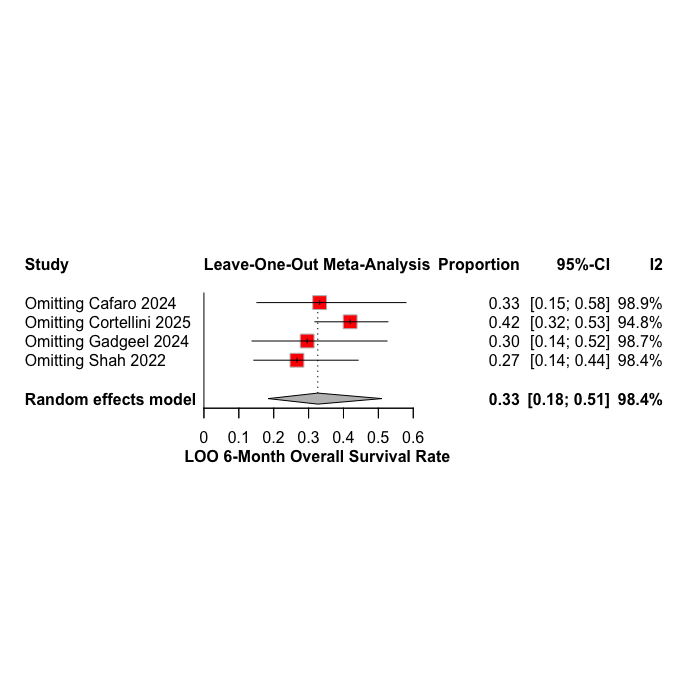

Supplement: Supplementary file 6 — Supplementary file6 (PNG 53 KB) [file 10434_2026_19138_MOESM6_ESM.png]

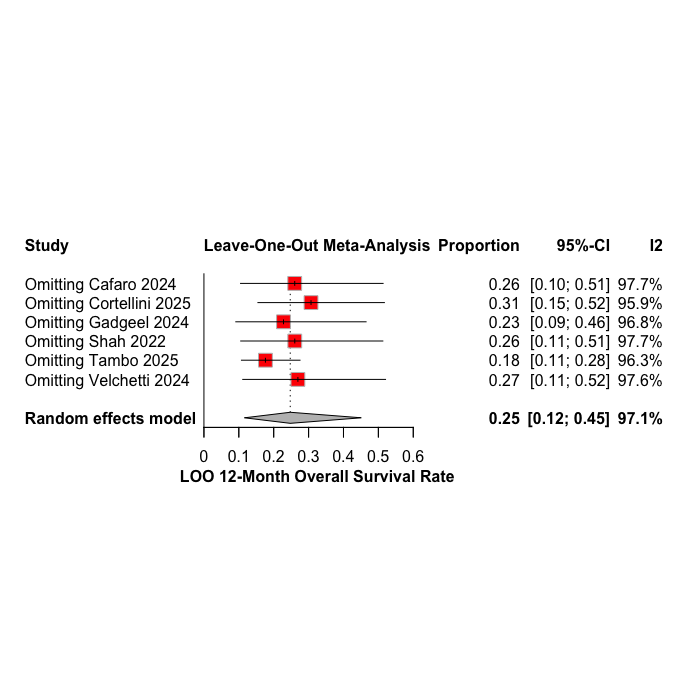

Supplement: Supplementary file 7 — Supplementary file7 (PNG 61 KB) [file 10434_2026_19138_MOESM7_ESM.png]

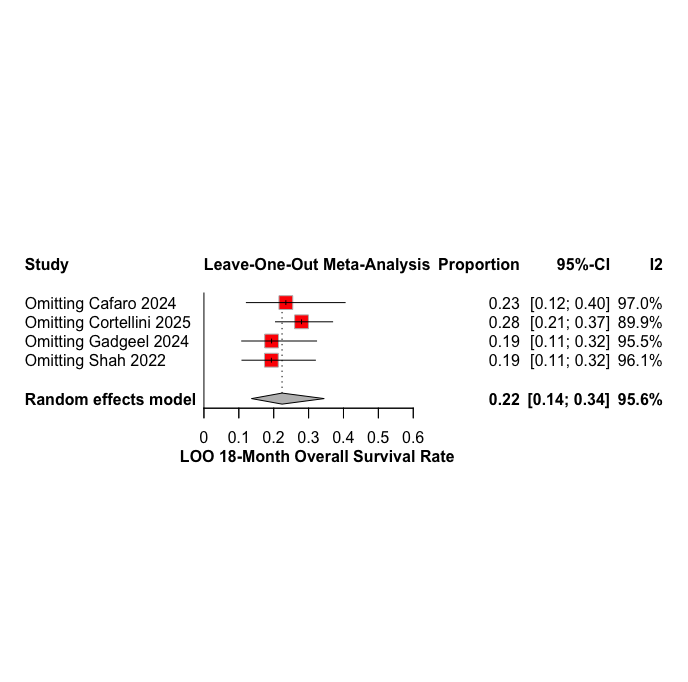

Supplement: Supplementary file 8 — Supplementary file8 (PNG 52 KB) [file 10434_2026_19138_MOESM8_ESM.png]

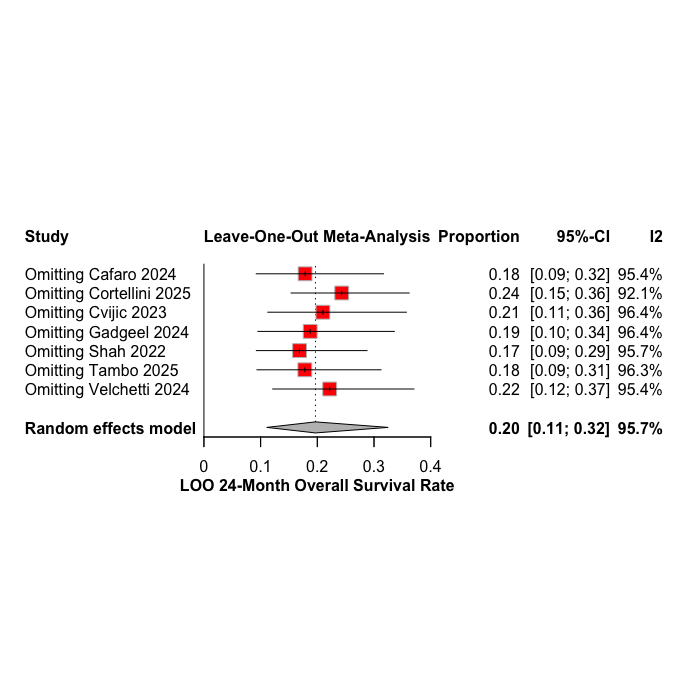

Supplement: Supplementary file 9 — Supplementary file9 (PNG 65 KB) [file 10434_2026_19138_MOESM9_ESM.png]

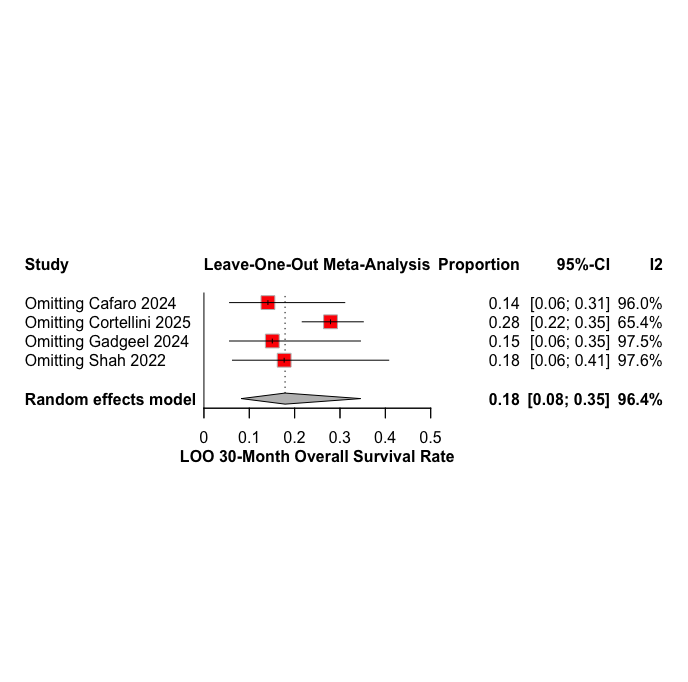

Supplement: Supplementary file 10 — Supplementary file10 (PNG 52 KB) [file 10434_2026_19138_MOESM10_ESM.png]

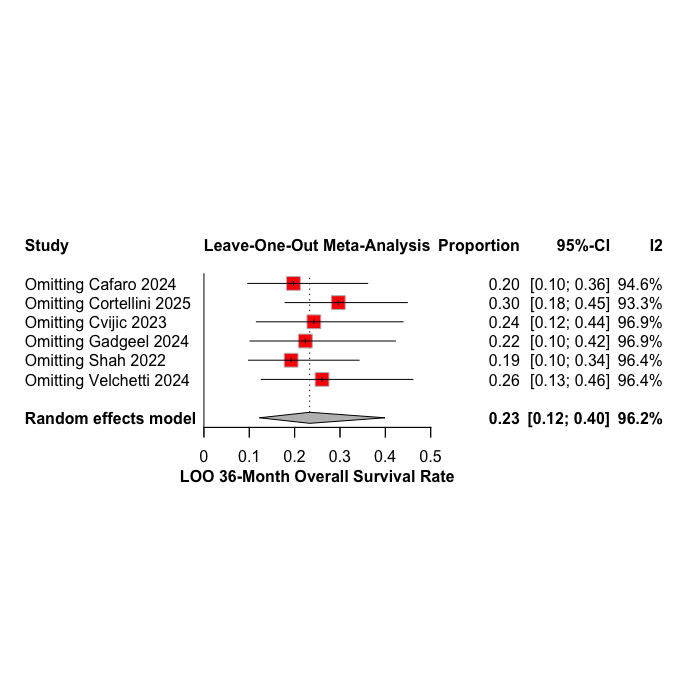

Supplement: Supplementary file 11 — Supplementary file11 (PNG 62 KB) [file 10434_2026_19138_MOESM11_ESM.png]

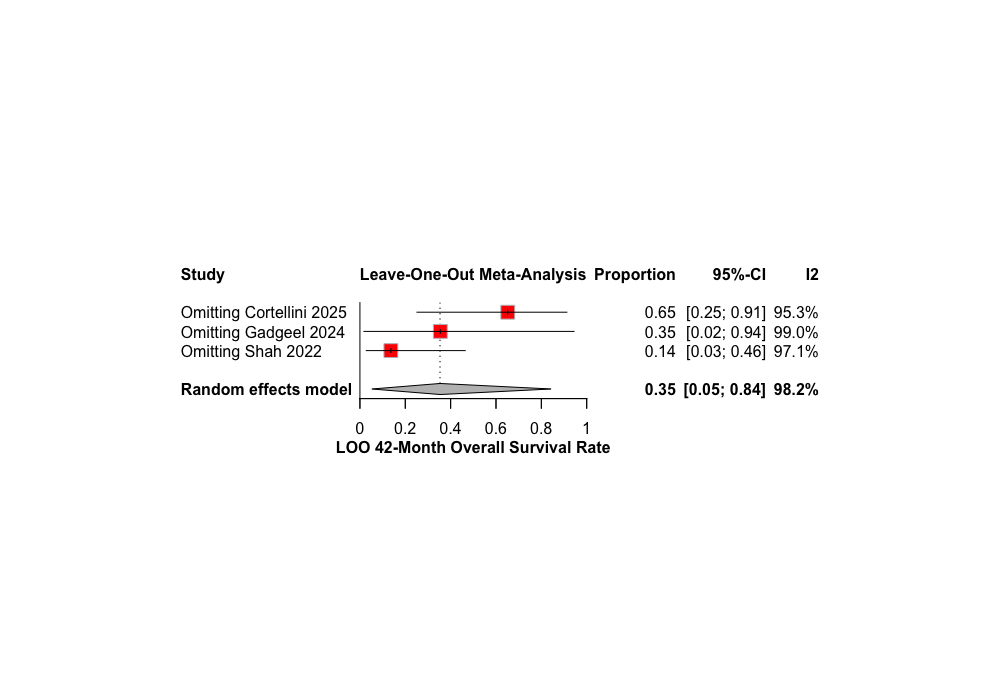

Supplement: Supplementary file 12 — Supplementary file12 (PNG 53 KB) [file 10434_2026_19138_MOESM12_ESM.png]

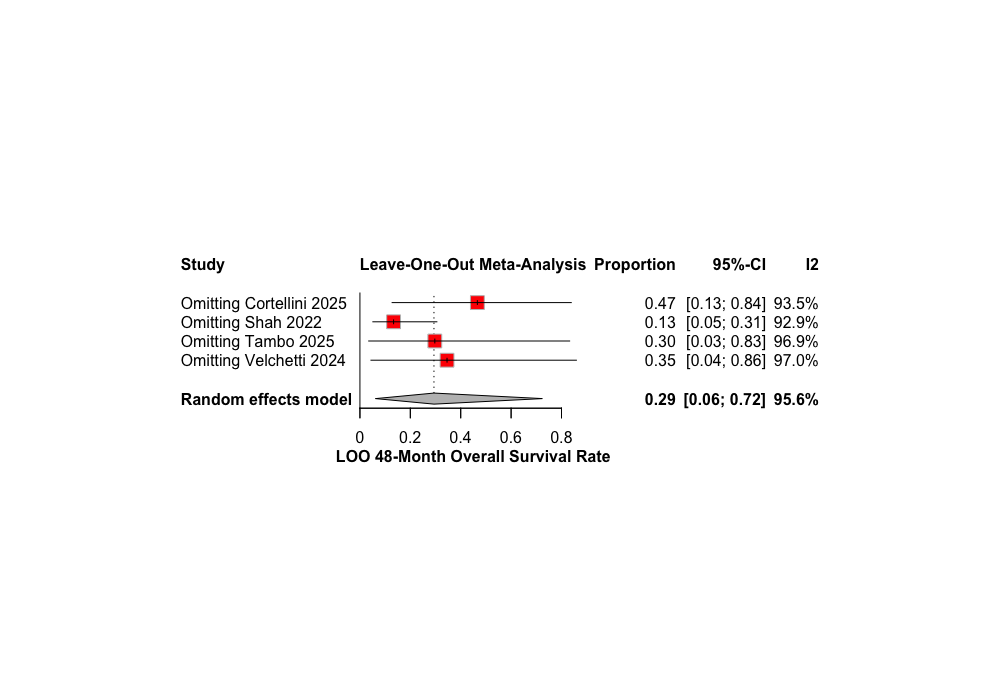

Supplement: Supplementary file 13 — Supplementary file13 (PNG 58 KB) [file 10434_2026_19138_MOESM13_ESM.png]

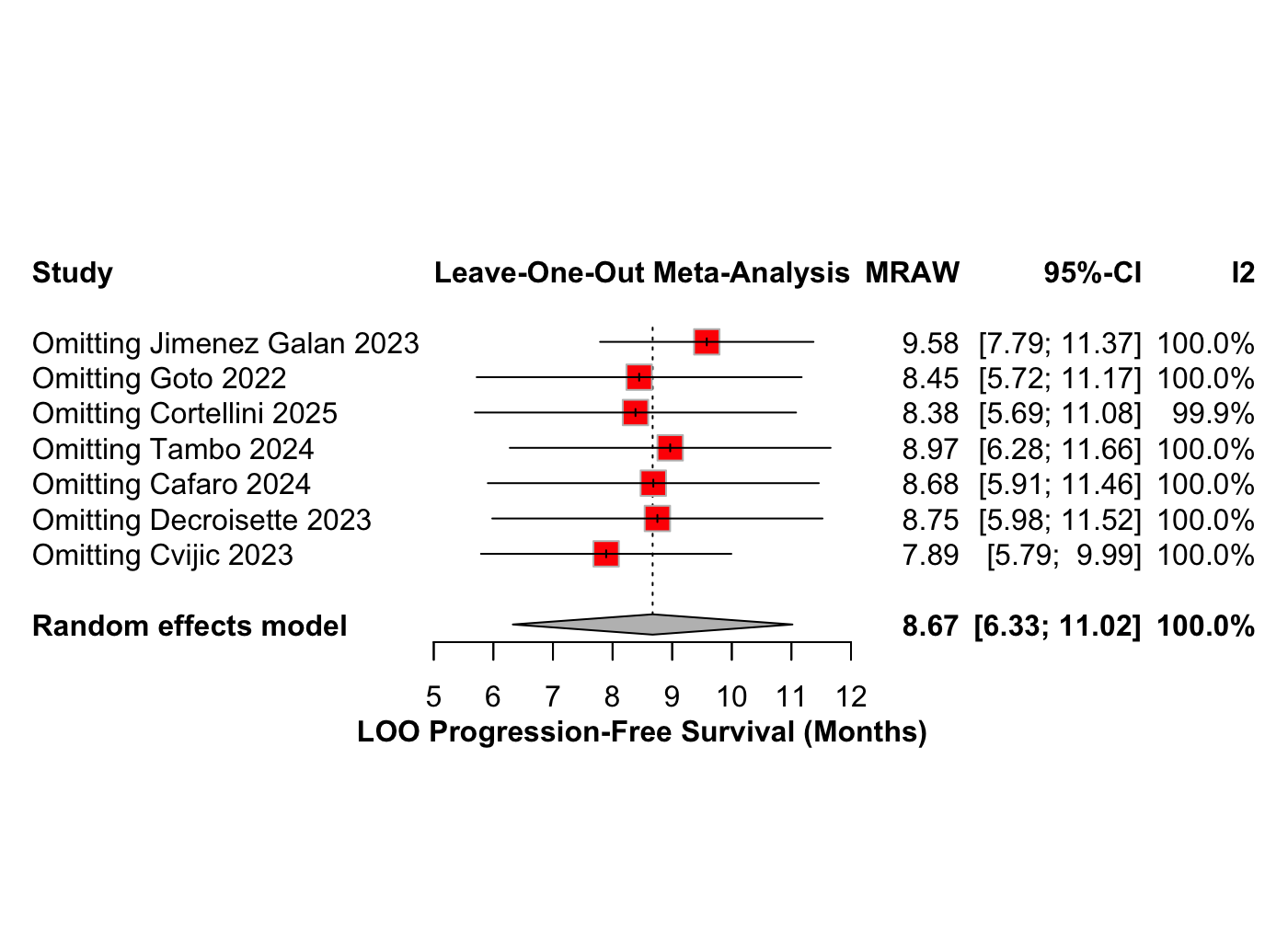

Supplement: Supplementary file 14 — Supplementary file14 (PNG 152 KB) [file 10434_2026_19138_MOESM14_ESM.png]

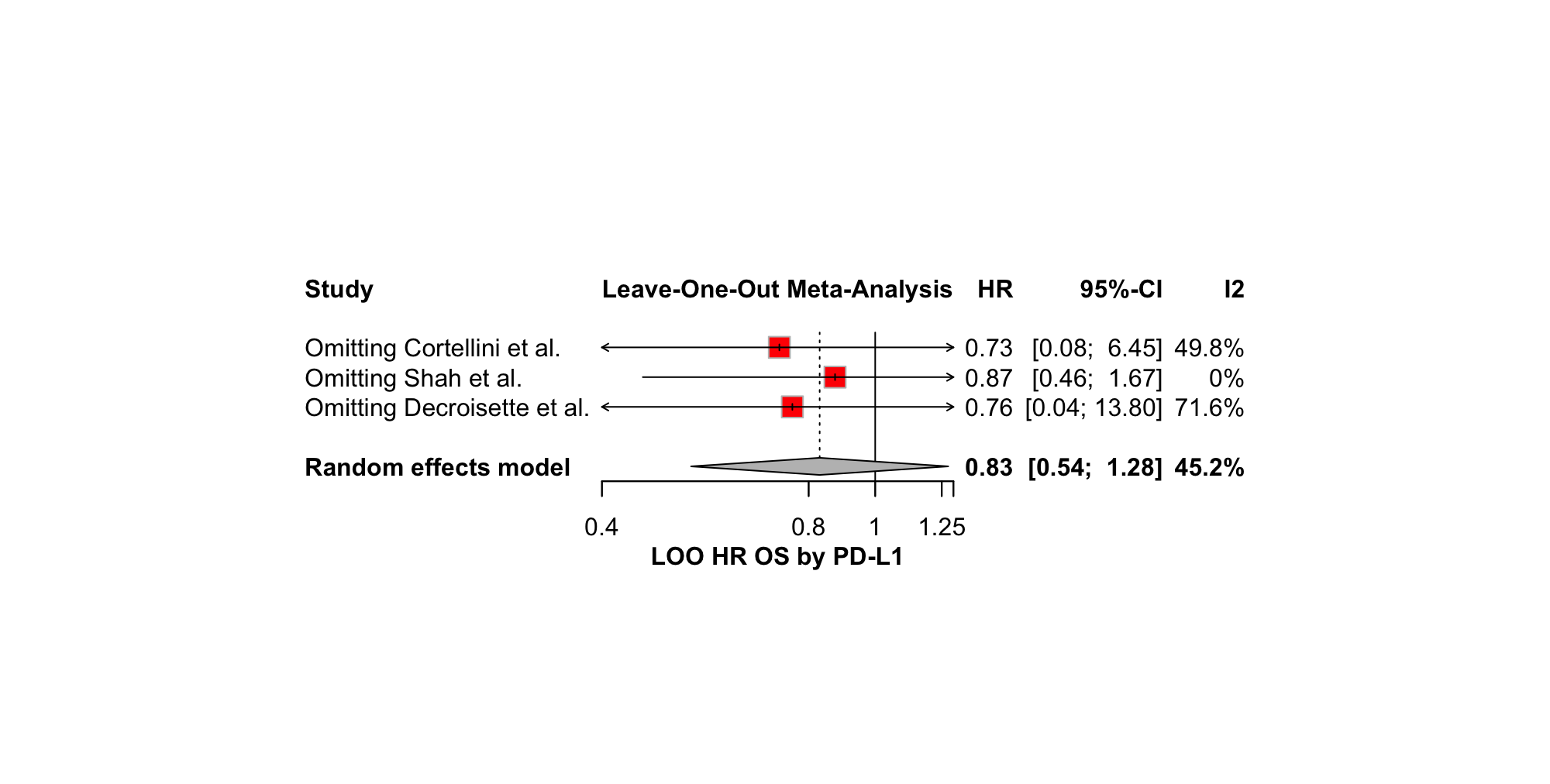

Supplement: Supplementary file 15 — Supplementary file15 (PNG 110 KB) [file 10434_2026_19138_MOESM15_ESM.png]

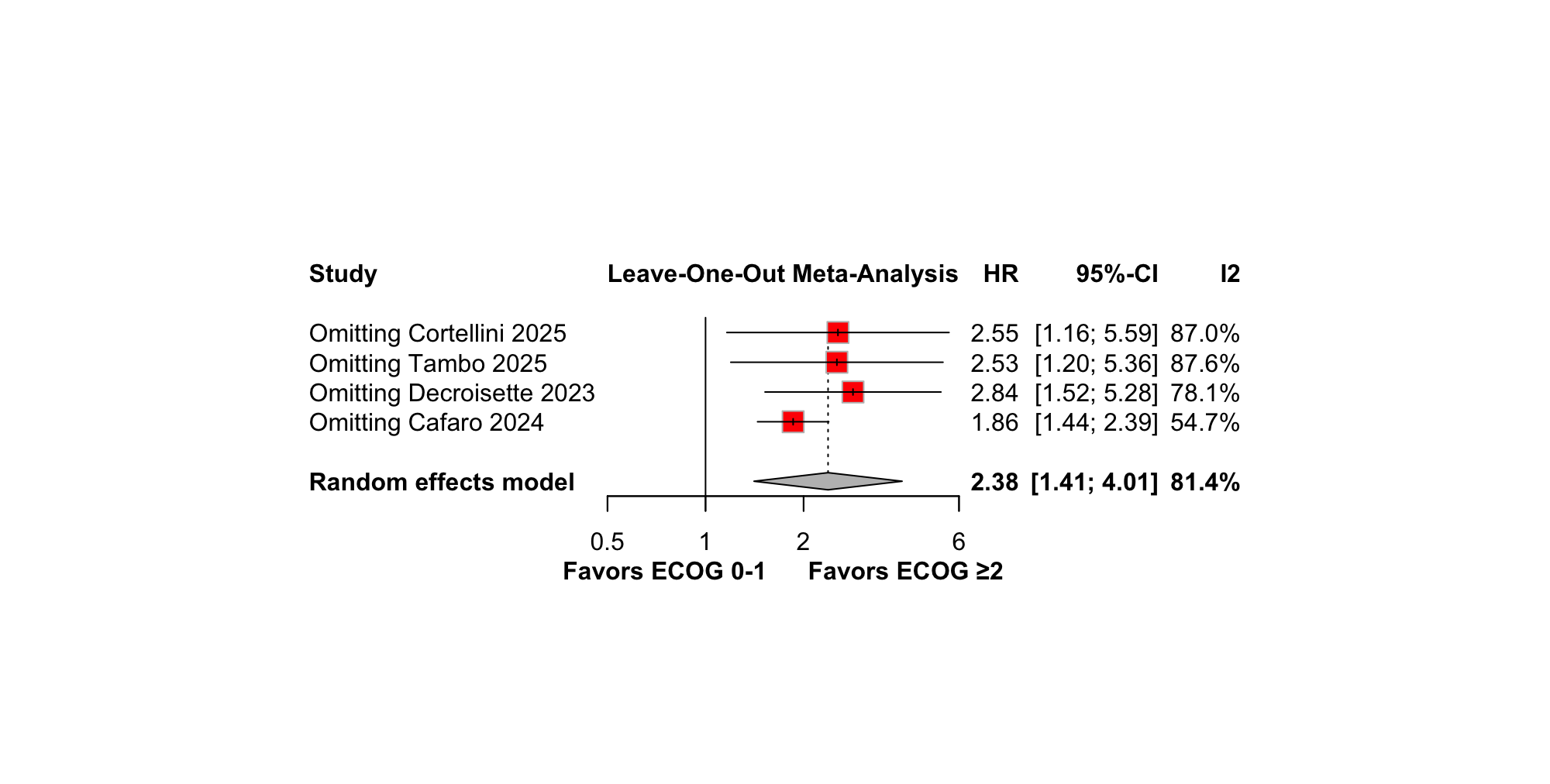

Supplement: Supplementary file 16 — Supplementary file16 (PNG 127 KB) [file 10434_2026_19138_MOESM16_ESM.png]
